# Variations in inflammatory genes are associated with periodontitis

**DOI:** 10.1186/1742-4933-10-39

**Published:** 2013-10-01

**Authors:** Manuela Ianni, Giacomo Bruzzesi, Davide Pugliese, Elisa Porcellini, Ilaria Carbone, Antonio Schiavone, Federico Licastro

**Affiliations:** 1 Department of Experimental Diagnostic and Specialty Medicine, University of Bologna, Via San Giacomo 14, Bologna 40126, Italy; 2 Euro Gen Med Prevention, Bologna, Italy; 3 Dental Care Center, San Patrignano, Rimini, Italy

**Keywords:** Immunology, Pathology, Inflammation, Cytokines

## Abstract

**Background:**

Periodontitis is a multi-factorial disease and several risk-factors such as infections, inflammatory responses, oral hygiene, smoke, aging and individual predisposition are involved in the disease. Pathogens trigger chronic inflammation with cytokines release which in turn leads to the destruction of the connective and the teeth supporting bone. The identification of genetic factors controlling oral inflammation may increase our understanding of genetic predisposition to periodontitis.

Single nucleotide polymorphisms in the promoter region of Vascular Endothelial Growth Factor, Alpha-1-Antichymotripsin, hydroxy-methyl-glutaryl CoA reductase, Interferon alpha, Interleukin-1 Beta, Interleukin 10, Interleukin 6 and Tumor Necrosis Factor- alpha genes from a case/control study were investigated.

**Results:**

The C allele of Vascular Endothelial Growth Factor, A allele of Interleukin 10 and GG genotype of Tumor Necrosis Factor-α were individually associated with chronic periodontitis. However, the concomitant presence of the three genetic markers in the same subjects appeared to play a synergistic role and increased several folds the risk of the disease.

**Conclusions:**

Our findings offer new tools to implement the screening of unaffected subjects with an increased susceptibility of periodontitis and increase our understanding regarding the genetic inflammatory background related to familiarity of the disease.

## Background

Periodontitis is a multi-factorial disease and several risk-components, such as environmental, metabolic, genetic, microbial factors, aging and poor oral hygiene status are involved in the pathogenesis of the disease [1].

Colonization of endogenous gram-negative periodontal bacteria including *Porphyromonas gingivalis*, *Aggregatibacter* (Actinobacillus) *actinomycetemcomitans*, *Tannerella forsythia* (forsythensis) and *Treponema denticola* appears to be the primary initiator of disease [2] and activator of abnormal chronic inflammation. It has been initially suggested that susceptibility to periodontitis could be genetically determined by the immune responsiveness to bacterial lipopolysaccharides [3]. However, since LPS is not the only bacterial products involved in periodontal inflammation, the genetic background of susceptibility to periodontitis remains largely to be determined.

Moreover, a variable degree of decline in the immune system efficiency is associated with aging and leads to an increased susceptibility of infections in the elderly. The periodontal apparatus is more vulnerable to destruction in aged individuals and immune senescence may contribute to periodontal disease of elderly [4].

Chronic inflammation and cytokines have been suggested to play a pivotal role in destructive processes occurring in periodontitis [5]. On the other hand, chronic periodontitis is associated with systemic disease where altered control of inflammation may play a role. In particular, chronic periodontitis may slightly influence the risk of cardiovascular disease, respiratory infections, adverse pregnancy outcome, rheumatoid arthritis and diabetes mellitus [6]. Family history of aggressive periodontitis is not uncommon and siblings of affected probands show an increased risk of the disease [7]. Therefore, inherited altered regulation of inflammatory responses may contribute to the pathogenesis of the disease.

Reports regarding genetic polymorphisms associated with periodontitis are increasing and several studies have shown that different cytokines are involved in periodontitis. For instance, single nucletotide gene polymorphisms (SNPs) of interleukin (IL-) 1α, IL-1ß, IL-4, IL-6, IL-8 and IL-18 located in different regions of the mentioned cytokine genes have been shown to affect the risk of the disease in several populations [8-12]. However, conflicting results regarding the association of SNPs in several genes with periodontitis are on record [13].

IL-10 SNPs, located both in the promoter or exon regions of the gene, resulted associated with a lower risk of chronic periodontitis [14].

A strong association between Tumor Necrosis Factor Alpha (TNF-α) rs1800629 and generalized forms of periodontitis was found [15]. A TNF-α promoter SNP (-308) has also been associated with the development of the disease and aggressive periodontitis [16]. However, the association of IL-10 and TNF-α SNPs with periodontitis in a subsequent investigation was not confirmed [17].

It is important to note that genotype prevalence appears to vary by race and ethnicity of the population studied. Therefore, the association of SNPs in candidate genes with modulatory activities on inflammation and periodontitis remains an open problem.

Highest mean gingival crevicular fluid and serum Vascular Endothelial Growth Factor (VEGF) concentration increased with the disease severity and reductions in VEGF levels in both gingival crevicular fluid and serum samples after periodontitis treatment were reported [18]*.* Epithelial expression of VEGF A, C, D in gingival was detected and increased numbers of immune cells expressing VEGF-C were found after infection, along with IL-1β and TNF-α protein upregulation [19].

A case–control study to identify the association of candidate genes epistatic interactions between genetic risk factors and susceptibility to aggressive periodontitis by using parametric analysis and higher order interaction models [20] has shown that: 1) within 14 candidate genes chosen in scientific literatures selenoprotein S (SEPS1) and tumor necrosis factor receptor superfamily member 1B (TNFRSF1B), have a possible role in determining host individual susceptibility to the disease; 2) an association between IL-6 and Fc-γ receptor polymorphism with periodontitis; 3) no the association of IL-1 cluster gene polymorphism with aggressive periodontitis.

Here, we investigated the genotype and allele distribution of SNPs in the promoter regions of several genes with inflammatory modulatory activity such as VEGF, Alpha-1-Antichymotripsin (ACT), hydroxy-methyl-glutaryl CoA reductase (HMG-CR), Interferon Gamma (INF-γ), IL-1ß, IL-10, IL-6, and TNF-α from ethnical homogeneous young patients with periodontitis. These genes were selected since their differential but pivotal modulation upon inflammatory processes. Controls consisted of healthy elderly without history of periodontitis. These SNPs were selected according to our previous investigations showing that these cytokines were associated with the risk to developing other chronic and degenerative disease such as acute myocardial infarction [21] and Alzheimer’s disease [22].

The aim of this study was to investigate the association of SNPs of several genes with immune regulatory function with periodontitis. Here we report a genetic signature that may be used to better assess the risk of the disease in unaffected subjects.

## Results

A panel of 8 SNPs in VEGF, ACT, HMG-CR, INF-γ, IL-1β, IL-10, IL-6 and TNF-α genes in patients with periodontitis and controls (CTR) was studied and genotype distributions and allele frequencies were obtained.

The genotype distribution and allele frequency in the promoter region of the -2578 C/A SNP in the VEGF gene are shown in Table 1. The CC genotype was more frequent in patients affected by periodontitis than CTR (72.7% vs 33.6%, p = 0.0001; OR = 5.259; ci = 2.956-9.357). The percentage of VEGF C carriers was significantly higher in the patients with periodontitis than in the CTR (94.8% vs 84.6%, p = 0.021; OR = 3.327; ci = 1.138-9.726), whereas the percentage of A carriers was significantly lower in the patients with periodontitis than CTR (27.3% vs 66.4%, p = 0.0001; OR = 0.190; ci = 0.107-0.338).

**Table 1:** VEGF, IL-10 and TNF-α genotype and allele frequency in patients with chronic periodontitis and CTR

**VEGF**	**CC**	**CA**	**AA**	**C carriers**	**A carriers**
	**(n) %**	**(n) %**	**(n) %**	**(n) %**	**(n) %**
**Periodontitis (n = 77)**	(56) 72.7	(17) 22.1	(4) 5.2	(73) 94.8	(21) 27.3
**CTR (n = 214)**	(72) 33.6	(109) 50.9	(33) 15.4	(181) 84.6	(142) 66.4
**Periodontitis vs CTR***χ*^2^ = 35.210 p = 0.0001;					
CC carriers *vs* non-CC carriers: *χ*^2^ = 35.104, p = 0.0001, OR = 5.259, ci = 2.956-9.357;					
AA carriers *vs* non-AA carriers: *χ*^2^ = 5.335, p = 0.021, OR = 0.301, ci = 0.103-0.879;					
C carriers *χ*^2^ = 5.335, p = 0.021; OR = 3.327; ci = 1.138-9.726;					
A carriers *χ*^2^ = 35.104, p = 0.0001; OR = 0.190, ci = 0.107-0.338.					
**IL-10**	**GG**	**GA**	**AA**	**G carriers**	**A carriers**
	**(n) %**	**(n) %**	**(n) %**	**(n) %**	**(n) %**
**Periodontitis (n = 75)**	(7) 9.3	(29) 38.7	(39) 52	(36) 48	(68) 90.7
**CTR (n = 470)**	(93) 19.8	(209) 44.5	(168) 35.7	(302) 64.3	(378) 80.4
**Periodontitis *vs *CTR***χ*^2^ = 8.850, p = 0.012;					
GG carriers *vs* non-GG carriers: *χ*^2^ = 4.870, p = 0.027, OR = 0.412, ci = 0.183-0.926;					
AA carriers *vs* non-AA carriers: *χ*^2^ = 7.054, p = 0.008, OR = 1.929, ci = 1.181-3.151;					
A carriers *χ*^2^ = 4.563, p = 0.033; OR = 2.364, ci = 1.051-5.318;					
G carriers *χ*^2^ = 7.255, p = 0.007; OR = 0.513, ci = 0.314-0.839.					
**TNF-α**	**GG**	**GA**	**AA**	**G carriers**	**A carriers**
	**(n) %**	**(n) %**	**(n) %**	**(n) %**	**(n) %**
**Periodontitis (n = 75)**	(68) 90.7	(7) 9.3	(0) 0	(75) 100	(7) 9.3
**CTR (n = 440)**	(349) 79.3	(87) 19.8	(4) 0.9	(436) 99.1	(91) 20.7
**Periodontitis *vs *CTR***χ*^2^ = 5.527, p = 0.063.					
GG carriers *vs* non-GG carriers: *χ*^2^ = 5.014, p = 0.025; OR = 2.463, ci = 1.094-5.548;					
AA vs non-AA carriers: *χ*^2^ = 0.861, p = 0.650.					
A carriers *χ*^2^ = 5.356, p = 0.021, OR = 0.395, ci = 0.175-0.889.					
G carriers *χ*^2^ = 0.687, p = 0.407.					

Table 1 also shows genotype distribution and allele frequency of the -1082 G/A SNP in the promoter region of IL-10 gene. The GG genotype was more frequent in CTR than in the patients with periodontitis (19.8% vs 9.3%, p = 0.027; OR = 0.412; ci = 0.183-0.926), on the other hand the AA genotype was more represented in patients than in CTR (52% vs 35.7%, p = 0.008; OR = 1.929; ci = 1.181-3.151). The G allele was significantly more frequent in CTR than in patients (64.3% vs 48%, p = 0.007; OR = 0.513; ci = 0.314-0.839) whereas the percentage of A carriers was lower in the CTR than in patients with periodontitis (80.4% vs 90.7%, p = 0.033, OR = 2.364; ci = 1.051-5.318).

The genotype distribution and allele frequency of the -308 G/A SNP in the TNF-α gene are shown in Table 1. The percentage of GG genotype was higher in patients with periodontitis than CTR (90.7% vs 79.3%, p = 0.025; OR = 2.463, ci = 1.094-5.548); on the other hand, A carriers were higher among CTR than patients (20.7% vs 9.3%, p = 0.021, OR = 0.395; ci = 0.175-0.889).

The concomitant presence of the C allele of VEGF, the A allele of IL-10 and the GG genotype of TNF-α was also determined and resulted to be associated with an increased risk of periodontitis, as shown in Table 2. The genetic signature of these "triple SNP carriers" was more frequent in the patients with periodontitis than in the CTR (79.7% vs 34.8%), and the difference was highly statistically significant (*X*^2^ = 47.780, p = 0.0001, OR = 7.375; ci = 3.973-13.689). An increased frequency of the triple SNP carriers was also present in the subgroup of patients with chronic generalized periodontitis (*χ*^2^ = 37.745, p = 0.0001, OR = 7.031, ci = 3.551-13.924), as shown in Table 3.

**Table 2:** Concomitant presence of the triple genotype/allele signature in CTR and all patients with chronic periodontitis

**Triple genotype**	**Carriers**	**Non-carriers**
	**(n) %**	**(n) %**
**Periodontitis (n = 74)**	(59) 79.7	(15) 20.3
**CTR (n = 276)**	(96) 34.8	(180) 65.2
**Periodontitis *vs *CTR***χ*^2^ = 47.780, p = 0.0001, OR = 7.375, ci = 3.973-13.689.		

**Table 3:** Concomitant presence of the triple genotype/allele signature in CTR and patients with chronic generalized periodontitis

**Triple genotype**	**Carriers**	**Non-carriers**
	**(n) %**	**(n) %**
**Generalized Periodontitis (n = 57)**	(45) 78.9	(12) 21.1
**CTR (n = 276)**	(96) 34.8	(180) 65.2
**Generalized Periodontitis *vs *CTR***χ*^2^ = 37.745, p = 0.0001, OR = 7.031, ci = 3.551-13.924.		

The SNPs in the promoter regions of the ACT, HMG-CR, INF-γ, IL-1β and IL-6 genes were also investigated, but no statistically significant difference in allele and genotype frequencies between CTR and patients with periodontitis was found (Table 4).

**Table 4:** ACT, HMC-CR, INF-γ, IL-1β, IL-6 genotype /allele frequencies in patients with chronic periodontitis and CTR

***ACT***	GG*	**GT**	**TT**	**G carr**	**T carr**
**Periodontitis**	(18)	(26)	(11)	(44)	(37)
(n = 55)	32.7	47.3	20	80.0	67.3
**CTR**	(108)	(222)	(122)	(330)	(344)
(n = 452)	23.9	49.1	27.0	73	76.1
*X*^2^ = 2.487 p = 0.288					
*X*^2^ = 1.238 p = 0.266 Allele G					
*X*^2^ = 2.049 p = 0.152 Allele T					
* wild type genotype					
***HMG-CR***	CC*	**CA**	**AA**	**C carr**	**A carr**
**periodontitis**	(56)	(8)	(2)	(64)	(10)
	84.8	12.1	3	97	15.2
(n = 66)					
**CTR**	(349)	(63)	(7)	(412)	(70)
(n = 419)	83.3	15	1.7	98.3	16.7
*X*^2^ = 0.915 p = 0.633					
*X*^2^ = 0.579 p = 0.447 Allele C					
*X*^2^ = 0.100 p = 0.752 Allele A					
* **wild type genotype**					
***IL-1β***	CC*	**CT**	**TT**	**C carr**	**T carr**
**periodontitis**	(34)	(29)	(8)	(63)	(37)
(n = 71)	47.9	40.8	11.3	88.7	52.1
**CTR**	(142)	(129)	(35)	(271)	(166)
(n = 306)	46.4	42.2	11.4	88.6	54.2
*X*^2^ = 0.052 p = 0.974					
*X*^2^ = 0.002 p = 0.968 Allele C					
*X*^2^ = 0.106 p = 0.745 Allele T					
* **wild type genotype**					
***INF-γ***	TT*	**TA**	**AA**	**T carr**	**A carr**
**periodontitis**	(16)	(38)	(14)	(53)	(52)
(n = 68)	23.5	55.9	20.6	79.1	77.6
**CTR**	(73)	(102)	(67)	(174)	(170)
(n = 242)	30.2	42.1	27.7	71.9	70.2
*X*^2^ = 4.055 p = 0.132					
*X*^2^ = 1.397 p = 0.237 Allele T					
*X*^2^ = 1.407 p = 0.236 Allele A					
* **wild type genotype**					
***IL-6***	GG*	**GC**	**CC**	**G carr**	**C carr**
**periodontitis**	(40)	(25)	(12)	(64)	(36)
(n = 77)	51.9	32.5	15.6	84.2	47.4
**CTR**	(119)	(125)	(34)	(244)	(159)
					57.2
(n = 278)	42.8	45	12.2	87.8	
*X*^2^ = 2.329 p = 0.127					
*X*^2^ = 0.669 p = 0.413 Allele G; *X*^2^ = 2.666 p = 0.103 Allele C					
* **wild type genotype**					

## Discussion

Periodontitis are infectious diseases in which bacteria and other pathogens trigger chronic inflammatory and immune responses with release of cytokine and other inflammatory mediators that lead to tissue destruction [1]*.* Aging is associated with hystological and clinical alterations in oral tissues. For instance, epithelium thinning and diminished keratinization occur in aged gingival and cellular components of gingival connective tissue decrease with age. These functional and structural alterations in gingival tissues along with a decline of the immune responses may result in an increased susceptibility of the elderly to microbial infections [4].

The gingival fibroblasts being constantly affected by the oral bacteria and their products like lipopolysaccharide, release inflammatory cytokines like prostaglandin E2, interleukin (IL)-1-β, and plasminogen activator [3,4] and these pro-inflammatory compound and cytokines may contribute to periodontal destruction [23,24].

Periodontitis is a complex, multifactorial and partially genetically determined disease and genes of the inflammatory response may modulate the periodontitis clinical expression [1]*.* Environmental risk factors by interacting with host genes play an important role in regulating the inflammatory response [1]*.*

It is interesting to note, that genome wide association (GWA) studies have shown an association of genetic loci located in chromosome 9 and 19 with severe chronic periodontitis [25]. Another GWA investigation reported that glycolyl-trasferase gene (GLT6D1) was a susceptibility locus for the disease [26].

Here, SNPs in genes with regulatory effects on inflammatory responses as possible genetic markers of periodontitis have been investigated. We selected ACT, HMG-CR, INF-γ, IL-1β, IL-6, VEGF, IL-10 and TNF-α genes, since they have relevant and differential role in regulating immune responses and have been found to affect the risk of other chronic diseases where immune alterations were linked to pathogenetic mechanisms [21,22,27].

SNPs in the promoter region of these genes have been selected, since variations in this region affect the synthesis of the cognate molecule [21,22].

Some studies have shown that high gingival crevicular fluid and serum levels of VEGF were associated with the severity of periodontitis [18]. However, as far as we know no data regarding SNP distribution in the promoter region of VEGF in patients affected by periodontitis have been reported. Our findings show that the CC genotype and the C allele of VEGF are more frequent in the patients with periodontitis than in CTR and result as genetic risk factors for the disease. Our findings suggest that homozygous subjects (CC) show a slightly higher risk of the disease than heterozygous (OR = 5.259 and OR = 3.327, respectively).

Lymphangiogenesis, the formation of new lymphatics, is associated with chronic inflammation and tissue injury; its role being to enhance lymphatic flow, immune cell transport and antigen clearance. Gingival growth of lymphatic vessels occurs during development of periodontitis and the upregulation of VEGF is likely to be important for the growth of lymphatic vessels [19]. VEGF expression is a key regulator of physiologic and pathologic angiogenesis and subjects with the CC genotype or the C allele may be prone to aberrant angiogenesis and lymphangiogenesis that in turn may predispose to the periodontitis.

Our data show that the A allele of IL-10 is more frequent in patients with periodontitis than in CTR and is a genetic risk factor for the disease (OR = 2.364). IL-10 polymorphism has been associated with chronic periodontitis and our data are in accordance with other investigations showing that SNPs in the IL-10 gene are associated with an increased risk and progression of periodontitis [28]. These findings support the notion that subjects with defective IL-10 production (A carriers) are prone to develop chronic inflammation and periodontitis. In fact, IL-10, an anti-inflammatory cytokine, seems to attenuate periodontal tissue destruction through the induction of tissue inhibitors of metalloproteinases and osteoprotegerin, an inhibitor of osteoclastogenesis [29]*.* Our findings are also in agreement with a recent report showing an IL-10 SNP association with chronic and aggressive periodontitis [30].

However, other studies did not support a role of IL-10 SNP in periodontitis [17]. These discrepancies may be attributed to different selection criteria of patients and controls or their ethnic heterogeneity.

Our findings also show that the GG genotype of TNF-α is a genetic risk factor for periodontitis (OR = 2.463).

Present data regarding TNF-α SNP association with periodontitis are in accordance with other case-controls investigations [15,16] and support a role for TNF-α in chronic inflammation and bone remodeling, as shown for different diseases such as rheumatoid arthritis [31].

Other studies, however, were not able to confirm any association of TNF-α SNPs with the disease [32,33]. Once again, patient/control selection criteria and/or ethnic heterogeneity may explain conflicting results. On the other hand the above factors (selection criteria, ethnicity) along with the severity of the disease may partially explain why we were not able to find any association of periodontitis with other SNPs such as INF-γ, IL-1β, IL-6. Our findings are in fact at variance with other investigations showing that gene variations of INF-γ, IL-1β and IL-6 were associated with periodontitis [8,10,16].

The concomitant presence of C allele of the VEGF gene, the A allele of the IL-10 gene and the G allele of the TNF-α gene ("triple SNP signature") is highly increased in patients with periodontitis (OR = 7.375). This triple SNP signature is also over-represented in the group of patients with generalized periodontitis.

The age difference between control and patients with periodontitis was relevant; however, such a difference could not influence the genetic inheritance of SNPs in genes with immune regulatory functions and the aim of this investigation was to find possible differential distribution in some SNPs of these genes. Moreover, drug-abuse and different life styles between patients and CTR may differentially affect susceptibility to periodontitis but, it is unlikely that these factors may influence the genetic background associated with the risk of the disease.

## Conclusions

Periodontitis is influenced by environmental, metabolic, microbial, poor oral hygiene status and life-style habits, as well as aging.

In this pilot study young patients with an increased susceptibility to develop periodontitis because of their former drug-use and life-style were selected to search for candidate genes associated with the disease. CTR group consisted of healthy elderly without history of clinical periodontitis and other inflammatory diseases. These differences between cases and CTR may increase the probability to find SNP associated with the disease. On the other hand, drug-abuse and different life styles between patients and CTR indeed differentially affect susceptibility to periodontitis, however it is unlikely that these factors may influence the genetic background associated with the risk of the disease.

SNPs in genes with regulatory immune function were selected. The C allele of VEGF, a regulator gene of angiogenesis and lymphangiogenesis, the A allele of IL-10, an anti-inflammatory gene and the GG genotype of TNF, a gene involved in both inflammation and bone remodeling, were associated with an increased risk of periodontal disease. Moreover the concomitant presence of these three genetic markers highly increased the risk of the disease. Therefore, these three genes appear to play a synergistic role in the development of chronic periodontitis. The concomitant presence of these different genetic markers may be used to identify among unaffected subjects with positive familial history of chronic periodontitis those with elevated risk of developing the disease and, therefore, be used to implement periodontitis prevention or early intervention screenings. However it is important to stress that these preliminary data need to be confirmed in larger case/control investigations to further assess their role in this disease.

## Methods

### Patients and controls

The study involved 77 patients with periodontitis (mean age 30 ± 6 years, 60 male and 17 female) from "San Patrignano Community", a private residential community for the rehabilitation of drug users located in Northern Italy. Patients suffered chronic periodontitis: 58 patients were affected by chronic generalized periodontitis and 19 patients by chronic localised periodontitis. The drugs users are usually at increased risk of developing periodontitis. Therefore, these subjects, because of their increased susceptibility to the disease may provide a good model to investigate candidate genes associated with the periodontal disease.

Clinical examination was performed by two dentists and the clinical diagnosis of chronic periodontitis followed criteria of the 1999 Consensus Classification of Periodontal Diseases [33]. Each patient was also investigated by oral radiograms. Chronic generalized periodontitis was diagnosed when more than 30% of periodontal sites was affected and chronic localized periodontitis when less than 30% of periodontal sites was involved. Exclusion criteria included history or current manifestation of systemic diseases such as diabetes, cardiovascular diseases, hepatitis, HIV infection and autoimmunity, use of antiseptic and anti-inflammatory drugs for a period longer than a week during the three months preceding the enrolment and smoking (no smoking during at least one year preceding the enrolment).

The control population belonged to the Conselice Study of Brain Aging from Northern Italy [21] and included 452 healthy subjects (mean age 72 ± 6 years, 235 male and 289 female) with no history of periodontitis and systemic diseases such as diabetes, cardiovascular diseases, hepatitis, HIV infection, autoimmunity or other inflammatory diseases before and during a 5 years follow up.

This elderly control population was selected to minimize the presence of subclinical periodontal alterations in our controls. Both periodontitis patients and controls were ethnically homogenous, since they were caucasian and from a restricted region of northern Italy written consent forms for genetic tests from each patient and control were collected. CTR and patients with periodontitis showed a significant age difference , however, controls have been selected according their negative anamnestic records of clinical peroidontitis with the aim to minimize the presence of the disease during their life. Moreover, such an age difference could not influence the genetic inheritance of SNPs in the gene with immune regulatory functions.

Moreover, drug-abuse and different life styles between patients and CTR may differentially affect susceptibility to periodontitis, however it is unlikely that these factors may influence the genetic background associated with the risk of the disease.

For the reduced number of case this study case–control it should be considered a "pilot study" to extend in a future at a large number of patients.

The research protocol was approved by the ethical committee of San Patrignano Community, Coriano, Rimini, Italy.

### DNA extraction

Epithelial cells samples from the oral cavity were collected by buccal swabs (Epicentre Biothechnologies, Italy). DNA extraction from swab was performed adding 600 μl of 50 mM NaOH to brush and heating at 95°C for 5 minutes. After brush removal, 60 μl of 1 M Tris pH 8 were added. Genome DNA was obtained spinning the sample at 13.000 rpm for 1 minute.

DNA CTR samples were obtained from peripheral blood leukocytes, as previously described [27].

Though DNA samples from periodontitis patients were obtained by buccal swabs while those from CTR were obtained from peripheral blood leukocytes, the difference in DNA sources does not have any relevance as far as SNP genotype is concerned, since genotype variations are equally distributed in all nucleated cells.

### SNP Detection

The presence of SNPs in the promoter regions of the VEGF (-2578 C/A, rs699947), ACT (-51 G/T, rs1884082), HMG-CR genes (-911 C/A, rs3761740) and IL-6 (-174 G/C, rs1800795) was assessed by polymerase chain reaction (PCR)-based methods as previously described [27,34-36]. SNPs in the IL-1β (-511 C/T, rs16944), IL-10 (-1082 G/A, rs1800896), IFN-γ (+874 T/A, rs2430561) and TNF-α (-308 G/A, rs1800629) genes were detected by real-time PCR. The SNP-specific primers and probes were designed using the TaqMan genotyping assay (ABI, Foster City, CA) in a 25 μl total volume of BIORAD CFX 96 in accordance with the manufacturer’s instructions [21].

The present investigation was a double bind study, since operators did not know the diagnosis of each subject.

### Statistical analysis

The genotype distribution and allele frequency from CTR and periodontitis patients were compared by contingency tables and chi square (*χ*^2^) analysis. Odds ratio (OR) and confidence intervals (c.i.) were also calculated and statistical significance assessed by using the SPSS 11.01 software package (SPSS Inc, Chicago IL, USA). Statistical tests were two-sided, and significance was set at p <0.05. Bonferroni’s correction for multiple comparisons was also used.

## Abbreviations

(SNPs): Single nucletotide gene polymorphisms; (IL-): Interleukin; (TNF-α): Tumor Necrosis Factor Alpha; (VEGF): Vascular endothelial growth factor; (ACT): Alpha-1-Antichymotripsin; (HMG-CR): Hydroxy-methyl-glutaryl CoA reductase; (INF-γ): Interferon Gamma; (CTR): Controls; (GWA): Genome wide association. The concomitant presence of C allele of the VEGF gene, the A allele of the IL-10 gene and the G allele of the TNF-α gene ("triple SNP signature").

## Competing interests

The authors declare that they have no competing interests.

## Authors’ contributions

This paper was made possible by the effective contribution of each author; MI, EP, IC performed the DNA extraction, genotyping and statistical elaboration; GB, DP, AS collected the samples and performed the examination and diagnosis of the partecipants; MI and FL wrote the paper; FL was responsible for the study’s design and scientific coordination. All authors read and approved the final manuscript.
